# Brainwave Activity Localization, Mood Symptoms, and Balance Impairment in a Male South African Rugby Player With Persisting Symptoms After Concussion: A Case Report

**DOI:** 10.1002/ccr3.70197

**Published:** 2025-02-10

**Authors:** M. J. Lumb, N. Snegireva, A. M. Coetzee, K. E. Welman

**Affiliations:** ^1^ Department of Exercise, Sport & Lifestyle Medicine, Division of Movement Science & Exercise Therapy, the Movement Laboratory, Faculty of Medicine and Health Sciences University of Stellenbosch Stellenbosch Western Cape South Africa

**Keywords:** athlete, case report, concussion, mobile EEG, qEEG analysis

## Abstract

The case sets the foundation for clinical protocols to incorporate mobile EEG and qEEG techniques, instrumental balance testing, and mood symptom screening in athletes who have suffered a sports‐related concussion. The protocol provides a framework for clinicians to monitor a patient's recovery progress in terms of brainwave activity, general cognition, moods, and motor control. Objective data obtained through the protocol may assist in developing personalized treatment plans, improving follow‐up care, and identifying residual brain function deficits that may be missed in standardized clinical exams. Finally, this case highlights a need for more thorough communication and testing procedures that screen for mood symptoms and provide an opportunity for athletes to discuss their mental health after suffering from an SRC.

**Abbreviations**
ASR: artifact subspace reconstruction
BESS: Balance Error Scoring System
CoP: center‐of‐pressure
CTE: chronic traumatic encephalopathy
EEG: electroencephalography
ESS: Epworth Sleepiness Scale
FSS: Fatigue Severity Scale
GAD‐7: generalized anxiety disorder 7‐item questionnaire
HREC: Health Research and Ethics Committee
ICA: independent component analysis
MoCA: montreal cognitive assessment
MRI: magnetic resonance imaging
PHQ‐9: patient health 9‐item questionnaire
PSaC: persisting symptoms after concussion
qEEG: quantitative electroencephalography

## 1 Introduction

Persisting symptoms after concussion (PSaC) is a complex pathophysiological process that refers to individuals who do not recover within the typical time frame after concussion [1]. Most concussive symptoms tend to peak hours after injury and gradually improve over days [1]. However, it has been suggested that clinical symptoms contradict physiological recovery and pathological developments may persist [1, 2, 3, 4, 5]. This can be in the form of irregular electrical brainwave activity, metabolic imbalance, reduced oxygen consumption, and reduced cerebral blood flow, resulting in the potential increase in the risk of re‐injury, psychological disorders, musculoskeletal injuries, or the development of chronic traumatic encephalopathy (CTE) [6, 7]. Such persisting symptoms have been suggested to present in 10%–30% of people, depending on the specific population cohort and the time frames used to define it [8, 9, 10].

Currently, mental health outcomes in athletes who experience PSaC are unclear and require further detailed investigation [11]. The prevalence of anxiety and depression in elite male athletes participating in team sports is nearly 45% [12]. Repetitive sports‐related concussions (SRC) are thought to increase the risk of the development of mental health problems, cognitive impairment, and sensorimotor disruption [13]. Persisting symptoms after concussion have been associated with higher levels of disability and psychological distress [10]. In addition to this, associations between persisting symptoms and depressive moods have been established; however, the causal directionality of the association is still up for debate [10]. Further focus on mental health screening and identifying replicable biomarkers associated with PSaC may lead to better athlete care and management, reducing the risk of re‐injury and further pathological developments, especially in the form of mental health degradation [10].

Postural stability is a complex motor skill that incorporates the integration and processing of dynamic sensorimotor information, and sports‐related concussions are believed to result in the dysfunction of sensorimotor integration caused by disrupted neural connections and axonal shearing [14]. Due to this neural trauma, PSaC may present, resulting in impaired motor outputs and an increased risk of suffering further concussions and other injuries, potentially also resulting in increased mood‐related symptoms [14].

It has also been found that emotions can affect motor behavior and that they are important indicators of mental illness [15]. Postural stability reflects multiple levels of the nervous system functioning together [15, 16]. These systems develop simultaneously with the development of higher‐level brain structures, including the prefrontal cortex, basal ganglia, and cerebellum [15]. It is thought that gait and postural discrepancies reflect impaired function of the cortical and subcortical regions [15]. Since psychiatric variables, gait, and postural stability function within these higher‐level brain structures, one can suspect that mood disorders such as depression and anxiety may have a direct impact on the capacity of the musculoskeletal system to adjust to stimuli; therefore, potentially justifying further research to establish if correlations exist between all three variables [15, 16].

A noteworthy consideration for practitioners treating in a sporting environment is to consider that multiple concussions may cause cumulative impact on balance, mood disorders, and long‐term cognition due to the direct physical neuronal trauma sustained during high‐impact head collisions. This trauma can result in the impairment of the sensorimotor integration process, leading to prolonged symptomology such as mood disorders and motor control dysregulation. Studies have shown that more than one concussion increases the risk of the individual experiencing PSaC, including balance impairment, mood disorders, cognitive decline, and a higher risk of the development of chronic traumatic encephalopathy (CTE) [17, 18].

Strides are being made to identify a replicable biomarker through the use of electroencephalography (EEG) data in combination with advanced mathematical software‐assisted analysis (qEEG) to determine sources of irregular brainwave functionality as well as provide evidence of brain injury in athletes with PSaC [19, 20, 21, 22, 23]. In addition to this, instrumented postural sway techniques are being explored to potentially detect more subtle deviations in postural stability within the population. These subtle deviations generally go missed in subjectively scored tests such as the standard Balance Error Scoring System (BESS) [24, 25].

This article describes the case of an active 21‐year‐old male professional rugby player who had suffered multiple concussions and was presenting with PSaC along with mood symptoms (depressive moods and anxiety‐like symptom) and fatigue symptoms. He underwent neurological and mood disorder screening as well as EEG testing while performing instrumented postural tasks. The case highlights a potential need for detailed and continued screening of mood symptoms in the population. Furthermore, it highlights the potential need to discover a replicable biomarker to determine regions of the brain that are most impacted as well as to determine a cost‐effective replicable instrumental balance test to detect subtle deviations in postural stability.

## 2 Case History

The participant was a 21‐year‐old male professional rugby player who played in the position of flank. He weighed 104.5 kg and was 190 cm tall. At the time of testing, he had been involved with professional rugby for 3 years and had suffered four confirmed diagnosed concussions, with the last one occurring approximately 13 months prior to the date of testing. The mechanism of injury involved direct contact to the right side of the head just above the ear (i.e., temporal bone). The participant was on the defending side when the injury occurred. Initial impact occurred when the participant launched himself in for a tackle and the ball carrier stepped into him, catching his head with his knee. The mechanism of injury was detailed from the participant's perspective. After the latest concussion, the participant was advised not to partake in contact sport for ± 6 months while he underwent cognitive and physical rehabilitation in the form of memory and balance training. Rehabilitation was completed, and at the time of testing, he was actively partaking in competition. The participant expressed concerns for his future health and indicated that it was unlikely that he would continue rugby after the completion of his studies due to the fear that further injury may occur.

Further descriptive and medical history details can be found in Table 1.

**TABLE 1:** Descriptive data and medical history.

Participant medical history/screening tool	Score/descriptors	Interpretation
MoCA (A.U)	26	Typical cognitive function
ImPACT (A.U)	5	The participant scored on the following criteria: (1) Nervous/Anxious (1), (2) Difficulty concentrating (1), (3) Difficulty remembering (1), (4) Visual problems (2). The scale is scored on a 0–6 scale, with 0 being none and 6 being severe
PHQ‐9 (A.U)	5 (Scored 0 on question 9)	Mild depressive mood severity with no suicidal or self‐harm tendencies/thoughts. Question 9 refers to suicidal and self‐harm tendencies. If a patient scores on this question, it requires an immediate referral for further diagnosis The following was scored on: (1) Little interest or pleasure in doing things (several days), (2) Feeling down, depressed, or hopeless (several days), (3) Trouble falling asleep or staying asleep, or sleeping too much (several days), (4) Feeling tired or having little energy (several days), (6) Feeling bad about yourself‐or that you are a failure or have let yourself or your family down (several days)
GAD‐7 (A.U)	5	Mild anxiety moods The following was scored on: (1) Feeling nervous, anxious, or on edge (several days), (2) Not being able to stop or control worrying (several days), (3) Worrying too much about different things (over half the days), (6) Becoming easily annoyed or irritable (several days)
FSS (A.U)	44	Suggests that fatigue has a high level of impact
ESS (A.U)	5	Suggests that the patient may not be suffering with excessive daytime sleepiness
Weekly alcohol consumption	± 7 units a week	
Alcohol consumption 24 h before testing	1.2 units	10 mL/8 g of pure alcohol or 25 mL of whiskey or equivalent. The participant indicated he had a beer 24 h prior to testing. Low levels of alcohol consumption are generally metabolized within several hours after consumption [26]
Learning disabilities/ADHD	No	
Hearing aids or pacemaker	No	
Previous neuromusculoskeletal injury	Grade 2 hamstring strain (unspecified), right. February 2023.	
Prescribed medication to assist with mood symptom management	Yes (unspecified)	The pharmacological prescription was for the continued mood symptoms experienced

Abbreviations: A.U, arbitrary units; ADHD, attention deficit hyperactivity disorder; ESS, epworth sleepiness scale; FSS, Fatigue Severity Scale; GAD‐7, general anxiety disorder; ImPACT, immediate post‐concussion assessment and cognitive testing; MoCA, montreal cognitive assessment; PHQ‐9, patient health questionnaire.

### 2.1 Case Examination Procedure and Results

Descriptive data were collected using a variety of questionnaires (Table 1). Additionally, a 64‐lead mobile EEG recording was conducted simultaneously with an instrumented BESS test [2, 27]. The case report was written using the Consensus‐based Clinical Case Reporting (CARE) guidelines checklist where applicable [28].

A medical history questionnaire was implemented to build a comprehensive background of the participant. This was followed by the Epworth Sleepiness Scale (ESS), the Fatigue Severity Scale (FSS), The Patient Health 9‐item Questionnaire (PHQ‐9), the Generalized Anxiety Disorder 7‐item Questionnaire (GAD‐7), the ImPACT Post‐Concussion Symptom Scale, and the Montreal Cognitive Assessment (MoCA) version 8.3.

Quantitative balance readings were collected utilizing the APDM Mobility Lab System (Opal, APDM Inc. Portland, OR) with a wearable body sensor attached around the participants lumbar region, L5 vertebrae as reference [25, 29].

The EEG reading was conducted using a compact wireless EEG amplifier and recorder (LiveAmp 64, Brain Products GmbH, Gilching, Germany) combined with a 64‐channel spandex (ActiCAP) active fitted electrode cap (ActiCAP, Brain Products GmbH, Gilching, Germany). The electrodes were positioned using the international 10–20 system [30, 31]. Recording was conducted at a sampling rate of 500 Hz with impedance set at 25 k‐Ω (kΩ) [32]. Electroencephalogram (EEG) data were obtained using the BrainVision Recorder (Brainvision Recorder, Vers. 1.23.001, Brain Products GmbH, Gilching, Germany).

Baseline EEG measurements were collected prior to testing to identify how specific movement artifacts may emerge. These baselines included: (1) 60 s natural stance, relaxed state, eyes closed, no counting or rhythmic activity, (2) 60 s natural stance, relaxed state, eyes open, no counting or rhythmic activity, (3) 30 s jaw clench, (4) 30 s continuously blinking, (5) 30 s head nodding, (6) 30 s head turning, (7) 30 s single leg balance with non‐dominant foot tapping, (8) 30 s lifting hands on and off iliac crests, (9) 30 s rocking back and forth on feet (i.e., between forefoot and heel).

The two 60 s standing still with eyes closed and eyes open baselines were performed and utilized as reference for the balance tasks in the BESS during data analysis. The 30‐s tasks were used to identify similar deviations in the recording during the BESS which may present as artifacts.

The BESS test was performed three times. Each test consisted of six trials taking place on a firm surface and on a medium‐density balance pad. Each trial was 20 s in duration, with rest periods between each stance. All stances were performed eyes closed. Subjective errors and objective center of pressure (CoP) measurements were also recorded and compared to normative data (Table 3) [29, 33]. Each BESS trial was completed in a systematic order: i.e., (1) double stance firm, (2) single stance firm (non‐dominant leg), (3) tandem stance firm (non‐dominant leg behind), (4) double stance foam surface, (5) single stance foam (non‐dominant leg), (6) tandem stance foam (non‐dominant leg behind). EEG data was collected continuously throughout the 3 testing trials, with time between stances and trials removed during the analysis to create a continuous EEG recording.

The BESS was chosen as it is a valid, reliable, cost‐effective and commonly used tool in concussion management, and it forms part of the SCAT6 assessment protocol [4]. However, several limitations exist namely the subjective scoring, learning effects and reduced sensitivity in detecting long‐term balance issues in persisting symptoms after concussion [24, 27]. Additional quantitative measurements in the form of a lumbar spine accelerometer to assess postural sway and potentially capture more subtle deviations in postural stability. Studies have shown that instrumented assessments of balance can detect subtle impairment and imbalances that persist beyond what can be seen visually [24]. The combination of the already established BESS and portable accelerometer used may provide a cost‐effective testing protocol for the use in PSaC in sports outside of relying on laboratory‐based procedures which are often costly. Other alternatives include computerized dynamic pictography, tandem gait testing with dual tasking and sensory organization testing.

The EEG data obtained from BrainVision Recorder were analyzed using MATLAB Version R2023b and the EEGLAB toolbox v2023.1.

A processing pipeline was developed by consulting previous studies that shared similarities and through tutorial presentations provided by the Swartz Centre for Computational Neuroscience, Institute for Neural Computation, University of California San Diego [19, 21, 23, 34] (Appendix A).

**FIGURE 1:**
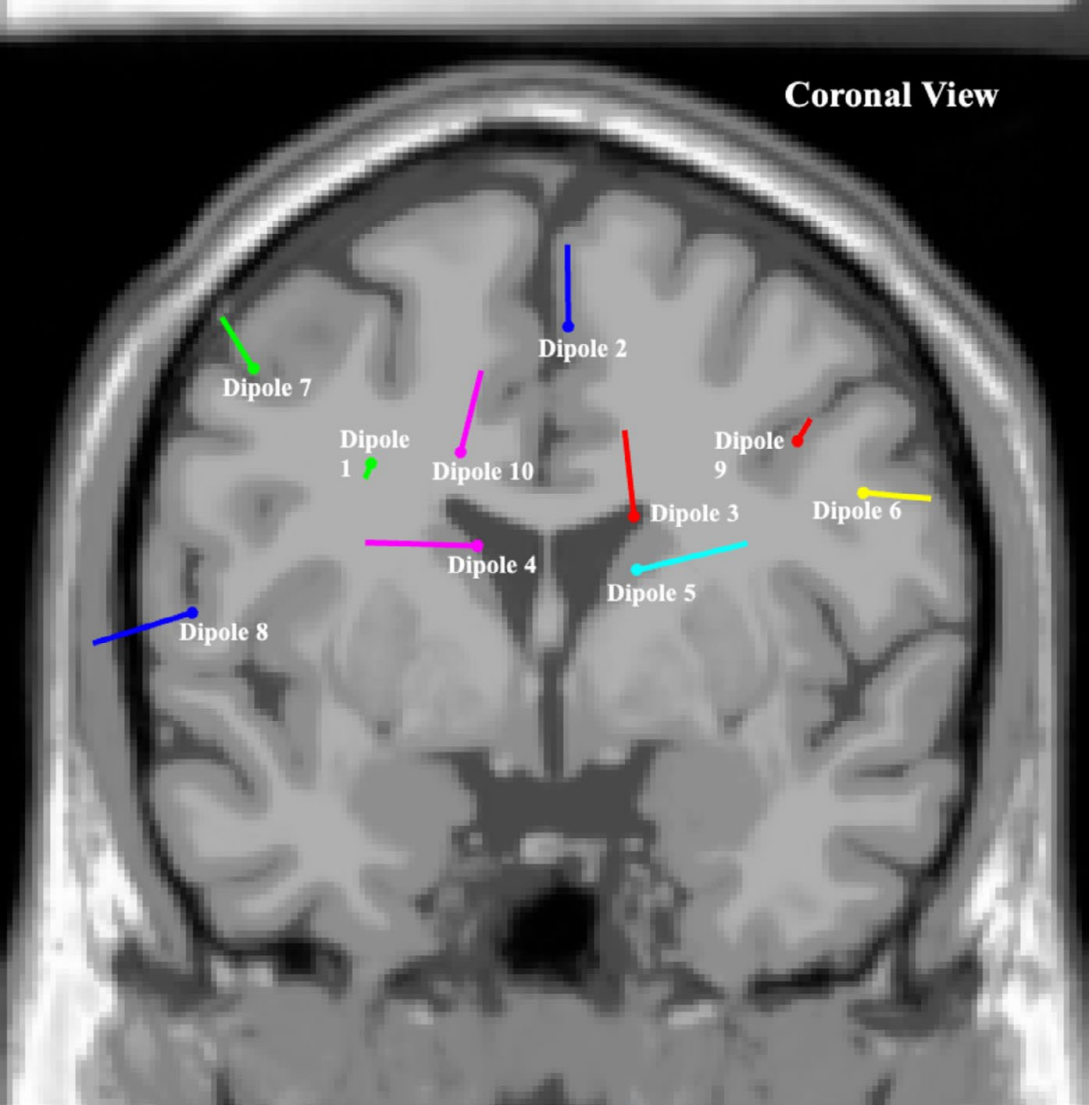
Coronal view of most significant dipole locations superimposed on an averaged referenced MRI.

**FIGURE 2:**
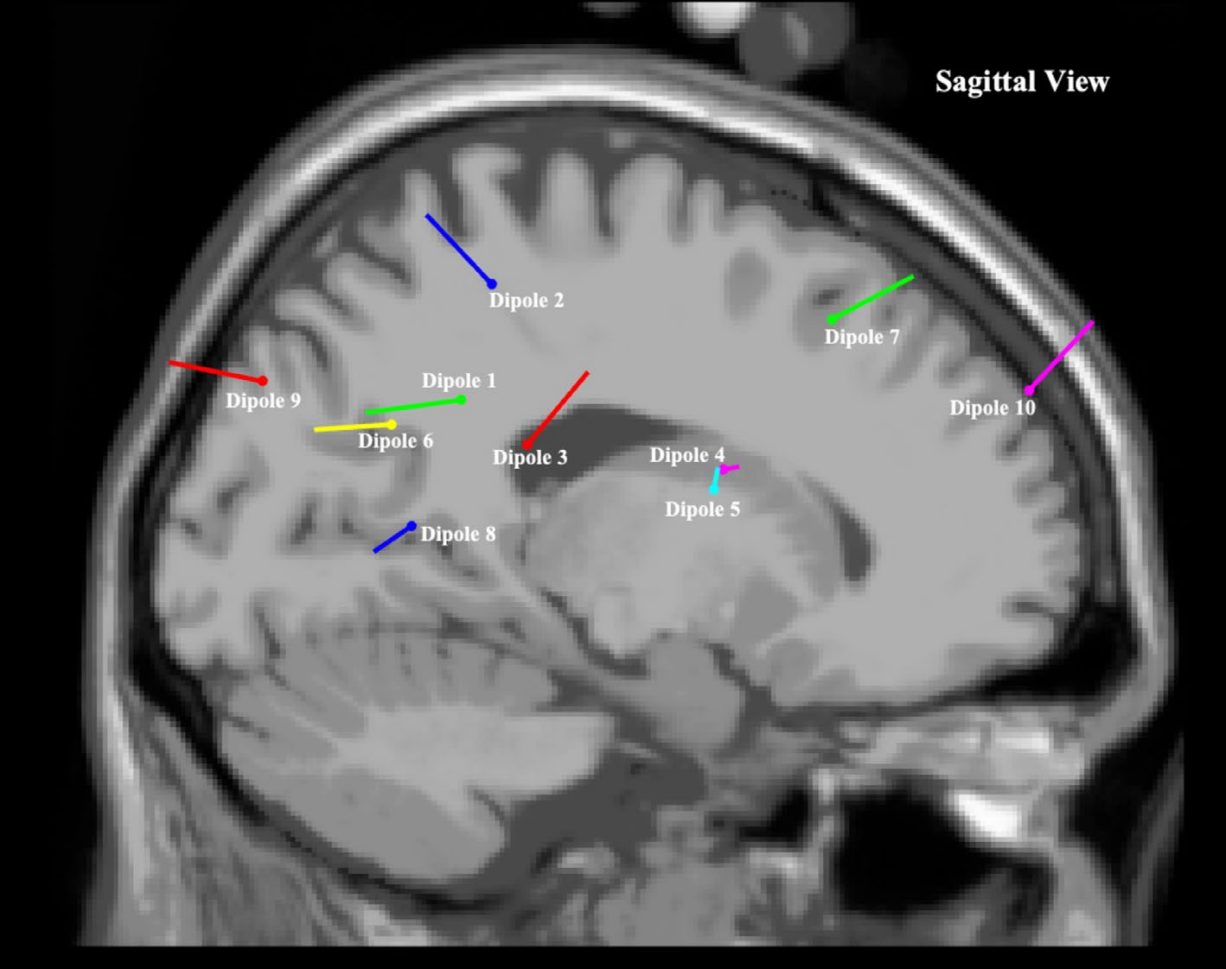
Sagittal view of most significant dipole locations superimposed on an averaged referenced MRI.

**FIGURE 3:**
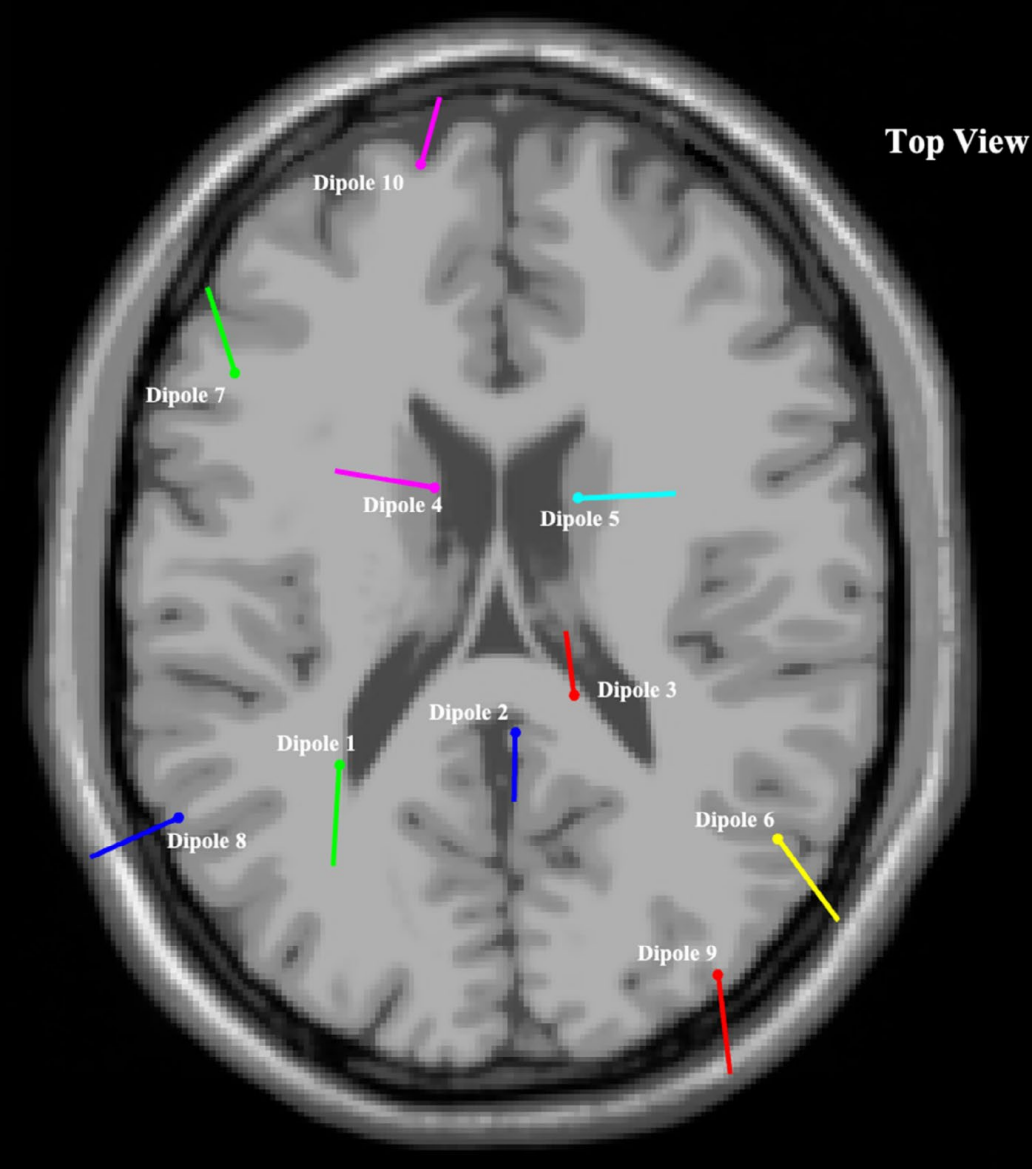
Top view of most significant dipole superimposed on an averaged referenced MRI.

**FIGURE 4:**
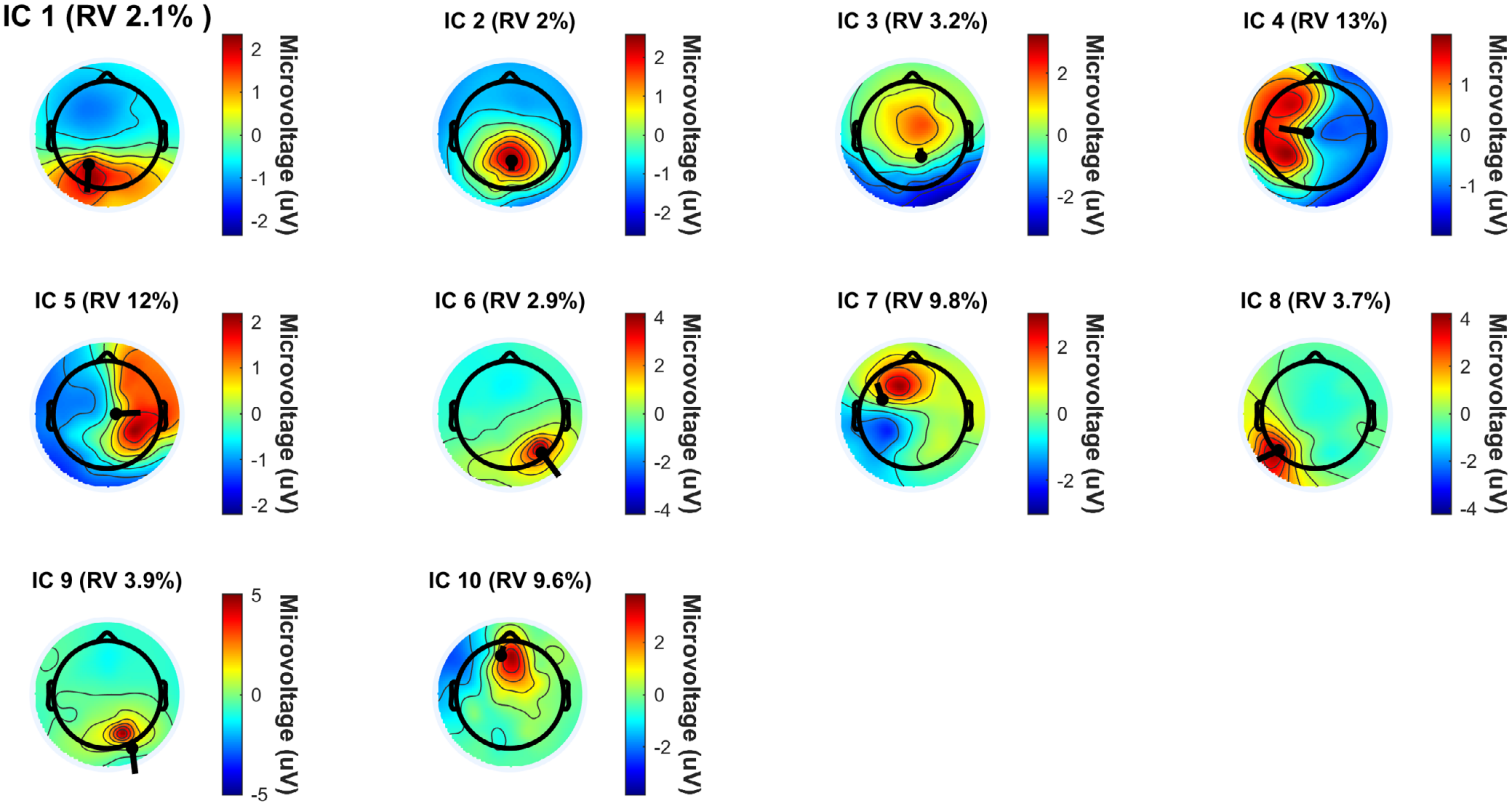
Topographical scalp maps of each identified individual component (IC) and Their corresponding dipole. The following topographical scalp maps visualize the distribution of electrical activity across the skull. The accompanying dipole represents the source localization of where suspected brainwave activity originated and the direction it traveled. Residual variance percentages (RV%) are also provided, indicating the error between the model's predicted brain activity and the actual recorded brain activity. A low RV% indicates that the model implemented explains the observed brainwave activity with a high level of confidence.

The most significant dipole locations that met inclusion criteria are depicted in Figures 1, 2, 3, with corresponding brain locations and coordinates presented in Table 2. Figure 4 displays the topographical scalp maps of each individual component analyzed, with the corresponding dipole location. Table 3 details subjective and objective BESS results.

**TABLE 2:** Component Brain Regions Using the Desikan‐Killiany Atlas and Talairach Coordinates.

Component/Dipole #	Closest brain region in Desikan–Killiany Atlas	Talairach co‐ordinates (X,Y,Z)	Residual variance (%)	General brain region's functions
1	Inferior parietal left	−29;−44;32	2.06%	Associated with spatial attention, sensory integration, and oculomotor control [35, 36]
2	Superior parietal right	3;−37;52	1.95%	Integral to visual, motor, cognitive, sensory, higher order cognition, working memory and attention processes [35, 36]
3	Superior parietal right	13;−32;23	3.22%	
4	Pre‐central left	−12;4;17	12.69%	Associated with voluntary motor control patterns [37, 38]
5	Post‐central right	14;2;14	12.49%	Contains the primary somatosensory cortex and is responsible for the integration of proprioception [39]
6	Inferior parietal right	50;−57;28	2.92%	Associated with spatial attention, sensory integration, and oculomotor control [35, 36]
7	Caudal middle‐frontal left	−47;25;42	9.79%	Plays a significant role in the development of literacy, and the process of reorientation to unexpected stimuli [40]
8	Lateral occipital left	−57;−54;10	3.66%	Contributes vastly to visual image processing and visual image communication with the cerebral cortex [41]
9	Lateral occipital right	39;−80;37	3.89%	
10	Superior frontal left	−14;61;28	9.64%	Key component in the neural network of working memory and spatial processing [42]

Abbreviations: #, number; %, percentage.

**TABLE 3:** Balance error scoring system results.

Subjective Score, Mean ± SD (A.U) [33]				
Stance/Measure	Firm surface error norm (A.U)	Foam error surface norm (A.U)	Participant average error firm (A.U)	Participant average error foam (A.U)
Double leg stance feet together	0.03 ± 0.26	0.13 ± 0.56	0	0
Single leg stance non‐dominant foot	2.50 ± 2.21	6.24 ± 2.36	0.33 ± 0.58	3.33 ± 0.58
Tandem stance	0.72 ± 1.15	2.90 ± 2.40	0	0

Objective Score, Mean ± SD (m/s^2^) [35]				
Stance	Firm surface RMS sway m/s^2^ norm	Foam surface RMS sway m/s^2^ norm	Participant 3 trial average RMS sway m/s^2^ firm	Participant 3 trial average RMS sway m/s^2^ foam
Double leg stance feet together	0.62 ± 0.40	N/A	0.12 ± 0.01	0.20 ± 0.03
Single leg stance non‐dominant foot	3.01 ± 1.52	N/A	0.28 ± 0.07	1.02 ± 0.52
Tandem stance	1.89 ± 1.28	N/A	0.13 ± 0.02	0.17 ± 0.05

Abbreviations: A.U., arbitrary units; N/A, not applicable; RMS, root mean square; SD, standard deviation.

## 3 Discussion, and Conclusion

### 3.1 Discussion

The current case set out to highlight the potential long‐term presentations of mood disorders that may present in rugby players with PSaC and have a history of SRC. Furthermore, it reports on areas of neuronal activity in the brain of the patient while performing balance tasks that were subjectively and objectively measured. Additionally, it presents a template that can be further developed to screen for mood‐related symptoms for the athlete as well as provide a safe opportunity for the athlete to communicate freely on how they feel.

This study incorporated screening for mood disorders in assessing PSaC, since trauma to the brain may have longer lasting effects in the form of anxiety and depressive symptoms, which in return may cause increased disruptions to postural stability and neuronal activity bidirectionally [15, 16].

Therefore, it was hypothesized that a complex multidirectional relationship exists between these variables, and it is believed that the findings from such a protocol would add to a better understanding of the brain–body connection related to balance and mood. These results may lead to the development of brain activity biomarkers, a detailed screening protocol for mood‐related disorders, and a quantitative postural stability test for diagnostic and prognostic purposes in a clinical environment for this population and possibly for others.

It is also worth noting that in this case the participant suffered multiple concussions that may have resulted in cumulative damage to the brain impacting balance, mood disorders, and long‐term cognition. This continuous trauma can result in the impairment of the sensorimotor integration process leading to prolonged symptomology such as mood disorders and motor control dysregulation [17, 18]. Studies have shown that more than one concussion increases the risk of the individual experiencing persisting symptoms including balance impairment, mood disorders, cognitive decline, and a higher risk of the development of chronic traumatic encephalopathy (CTE) [17, 18].

The novelty of this case lies in the fact that the EEG recording was collected during instrumented balance tasks and not during resting states [21]. Athletes require a complex brain–body connection to perform optimally at the highest level; therefore, ideally, the testing should mimic the systems that are involved during complex motor control tasks performed while playing sports. Postural sway testing is also used as a diagnostic course prediction tool for individuals with mood disorders and as a return‐toto‐play test in SRC, most noticeably in the SCAT6 [2].

#### 3.1.1 Brain Regions Associated With Sport‐Related Concussions and Persisting Symptoms

Sports‐related concussion research has indicated that the frontal lobes, anterior cingulate, cerebellum, and the parietal lobes are the most affected brain regions, similarly presenting in the current case (dipole 1, 2, 3, 6, 7, 10) (Table 2) [20, 43]. Furthermore, it has been suggested that diffuse axonal injury may occur in the hippocampus, resulting in long‐range communication deficits between it and other brain regions such as the prefrontal cortex and the amygdala [44]. These long‐range communication deficits have been found to persist after clinical symptoms have subsided, displaying abnormalities within central and primary white matter structures [45].

The current case displayed similar regions of neuronal activity when compared to previous studies, most noticeably in the left superior frontal cortical region (i.e., dipole 10) (Table 2) [21]. This brain region is thought to contribute most to higher cognitive functions such as working memory [46]. A previous study that utilized a form of ICA decomposition processing to clean EEG data and transform scalp sensory readings into brain sources found increased beta band power and decreased delta and theta band power in the frontal cortex in the concussed group [21]. This mirrors findings in previous fMRI studies showing changes in functional connectivity within frontal regions of the brain as well as findings in this case study where significant neuronal activity was found in the pre‐central left region (i.e., dipole 4), left superior frontal region (i.e., dipole 7) and the left middle frontal region (i.e., dipole10) [45].

It has been theorized that the above‐mentioned affected region, cognitive impairment, and slower neuronal processing speeds of stimuli in the brain are consequences of axonal injury, changes in neurotransmission, and irregularity in glycolysis homeostasis [44]. This may be due to the mechanism of injury associated with SRCs, namely, direct biomechanical contact forces or inertial forces leading to significant acceleration, deceleration, and/or rotational forces applied to these brain regions [4, 22, 44, 47].

#### 3.1.2 Mood Disorders Such as Depression and Anxiety in Persisting Symptoms After Concussion as Well as Brain Regions Associated With Mood Disorders

Although associations between mental health variables such as depression and anxiety have been suggested, the findings remain inconclusive, requiring further investigation possibly due to limited understanding [11, 48]. The importance of athlete mental health is rising due to the generic and sport‐specific stressors athletes are exposed to as well as the potential commonality between damaged regions of the brain due to the SRC and those associated with mood‐related symptoms [49, 50]. Experts have indicated that it is necessary for practitioners to recognize these symptoms early through thorough screening and implement graded exercise as well as education and counseling for the athlete [48].

The participants scores on the PHQ‐9, GAD‐7, and ImPACT (Table 1) align with current literature that indicates a bidirectional association between mood symptoms and individuals experiencing PSaC exist with those presenting with PSaC being at a higher risk of experiencing depressive and anxiety‐based symptoms, up to 10 years' post‐injury [10]. It is also worth noting that several other variables have been associated with the risk of developing PSaC, such as age (adolescents to young adults have been suggested to be at higher risk), the female sex, the mental health history of the athlete, and the number of SRCs suffered [10]. These are future areas that should be focused on by researchers in the context of SRCs and PSaC.

With regards to qEEG findings, the participant also presented with similar regions of neuronal activity when compared to previous studies that explored abnormalities in task‐related brain activation in patients with mood and anxiety disorders (i.e., dipole 1, 4, 6, and 10) (Table 2) [41, 51].

These studies had found hypoactivity in the frontal regions of the cortex, more specifically in the inferior prefrontal cortex/insula, the inferior parietal lobules, and the putamen, reducing engagement in inhibitory control and salience processing which could potentially impact motor control through the impairment of attention, awareness, sensory information integration, and executive functioning processes [51]. Hyperactivity had also been detected in the cingulate, amygdala, parahippocampal, and thalamus cortical regions suggesting over‐engagement of the regions of the brain associated with emotional regulation. Over‐allocation of neural resources to these may impact an individual's neural capacity to communicate and process stimuli impacting the bottom‐up (sensory‐driven) and top‐down (cognitive‐driven) neural communication mechanisms [51, 52].

Individuals with depressive moods have also displayed reduced function in the occipital lobes due to possible reductions and imbalances of neurotransmitters [41]. Similar brain regions in the participants were observed to be active, including dipole 8 and 9 (Table 2). Thus, this is a region of interest to compare to a group presenting with minimal to no mood disorders.

#### 3.1.3 Postural Control and Persisting Symptoms After Concussion as Well as Brain Regions Associated With Balance

This case did not present with subjective or objective balance scores outside of identified normative data (Table 3) [29, 33]. This may be due to greater motor control capacity in professional athletes resulting in fewer subjective errors and lower sway variations presenting during the BESS protocol. In addition, the results may have been impacted due to the participant having undergone physical rehabilitation treatment for the persisting symptoms and having already returned to play, potentially resulting in above‐average motor control capacity.

The current case did, however, present similarly to previous neural imaging studies that explored brain regions associated with balance and postural control, with regions of activity presenting in the frontal, parietal, and occipital brain regions (i.e., dipole 1–10) (Table 2) [53]. Nearly every region in the brain is thought to be involved in balance, with the cerebellum playing a pivotal role [53]. Previous studies also pointed to subcortical regions, namely the basal ganglia and thalamus, as important role‐players in balance [53].

It is therefore not surprising that neuronal activity was detected in these cortical brain regions due to the complex task of postural stability and motor control, especially when performed on an unstable surface, in unstable stances, and with a sensory (visual feedback) input removed [53]. In addition, neuronal activity in these regions may have been caused by a suboptimal decision‐making process due to disturbance of the neuronal capacity by depressive moods, anxiety‐like symptoms, and suspected long‐term axonal micro‐trauma [51].

The inferior parietal cortex has been associated with the planning and interpretation of sensory information. The current case displayed neuronal activity in this region (i.e., dipole 1 and 6) (Table 2) [53]. While not directly linked to motor execution, this region has been suggested to play a role in balance through higher‐order integration of information [53]. Findings in the parietal regions were also more common in studies that measured dynamic balance [53]. These findings were reflected in the current case report (i.e., dipole 1 and 6) (Table 2).

Frontal lobe, temporal, and occipital regions also displayed signs of activity in the current case report (dipole 4, 7, 8, 9, and 10) (Table 2); however, the variability across multiple structures within them indicates a need for further studies to explore their specific role in balance regulation [53].

Another stand‐out region of brain activity in the current case was the occipital lobe region (dipole 8 and 9) (Table 2). Since this region of the brain is most associated with visual imagery processing, the activity might be due to the fact that tests included a 60‐s eyes‐open natural double stance baseline (used for reference in qEEG data processing) [41].

### 3.2 Conclusion

One cannot make an inference from one case and further data collection; longitudinal and comparative studies (i.e., pre‐ and post‐concussion) should be conducted to determine if significant changes are present. Study limitations and future research suggestions are discussed in Appendix B.

## Author Contributions

**M. J. Lumb:** conceptualization, data curation, formal analysis, investigation, methodology, project administration, writing – original draft, writing – review and editing. **N. Snegireva:** conceptualization, supervision, writing – original draft, writing – review and editing. **A. M. Coetzee:** data curation, investigation, methodology, project administration, writing – review and editing. **K. E. Welman:** conceptualization, funding acquisition, resources, supervision, writing – review and editing.

## Ethics Statement

The study was ethically approved by the Health Research and Ethics Committee (HREC) under the following project ID: 26062 and reference number: S22/08/145 (PhD). Written informed consent was provided by the participant, providing permission for his findings to be published in academic journals as long as all identifiable indicators were removed. Original written consent forms will be retained by the primary researcher and provided to the publisher if requested.

## Conflicts of Interest

The authors declare no conflicts of interest.

## Data Availability

The raw data and relevant supporting materials for the study will be made available to other researchers upon request. Request for access to the data should be directed to the corresponding author. Raw data will be anonymised, and password protected before being shared.

## Appendix A qEEG Analysis Pipeline

Baseline tasks (excluding 60 s standing eyes open and eyes closed), rest periods between tasks, errors, and other artifact‐like activity were initially removed from the dataset [34]. A basic FIR filter was then applied with a high‐pass filter of 0.5 Hz and a low‐pass filter of 45 Hz [19, 21]. A high‐pass linear filter of 0.5 Hz was applied because independent component analysis (ICA) is very sensitive to lower frequency shifts, and the impacts of concussion and specifically those associated with persisting symptoms, display higher power in the delta band. A low‐pass linear filter of 45 Hz was chosen due to this being similar to previous studies that applied 40 Hz and 45 Hz, as well as the frequencies of interest in this study [19, 21]. Gamma band frequencies have been found to be most apparent around 40 Hz, and EEG spectrum analysis for concussion has found characteristic waveforms between 1 and 40 Hz [19]. The data were then down‐sampled to 100 Hz as brain activity is typically not quantified beyond this point, and spectral and spatial filtering does not perform well with higher sampling rates due to increases in data complexity [54].

Spectral and spatial filtering was applied using both Zapline‐plus (i.e., line‐noise frequencies specified at 50 Hz and automatic noise detection set to the defaulted value of 4) and Cleanline V2.00 (i.e., line‐noise frequencies specified at 50 Hz), scan for line noise set to “true”, detection of significant sinusoids set to *p* = 0.01, bandwidth scanning set to 2 Hz, taper bandwidth set to 2, sliding window length (sec) set to 4, sliding window step size (sec) set to 1, window overlap smoothing factor set to 100, and Fast Fourier Transform (FFT) padding factor set to 2. These line‐noise frequencies were specified as South Africa's electrical power grid is synchronized at 50 Hz; however, it is characterized as stochastic in nature, with dispatch changes and electricity demand resulting in large frequency deviations [54, 55, 56]. In addition to this, one needs to take into account sub‐harmonics (i.e., integer divisions and multiples of line noise frequency) that emerge with line‐noise frequencies; in the case of a 50 Hz power grid and a data filtering rate of 100 Hz, the former is deemed sufficient [54].

Clean_rawdata v2.91 plugin was used for automatic bad channel removal. Channels were removed if the following criteria were met: (1) were flat for more than 5 s, (2) had high‐frequency noise above 4 SDs, (3) and/or a minimum correlation between neighboring channels below 0.85 (*r* < 0.85). Artifact Subspace Reconstruction (ASR) was used to correct the continuous data recording of high‐amplitude artifacts; default settings were used [21, 57, 58].

Electrode channel locations was then aligned with the MNI coordinate file so to be fitted to the boundary element head model (BEM). The boundary element model (BEM) was selected over the spherical four‐shell model (BESA) as it is a more accurate model that will return more accurate dipole positions superimposed onto a more detailed MRI image [59].

A zero‐filled channel was then added back into the dataset, averaged referenced, and then removed. This was done to avoid ghost components emerging during ICA due to the presentation of effective rank‐deficient data caused by EEG electrode interpolation and inadequate re‐referencing methods that are applied in EEGLAB's re‐referencing processes [60].

Data were then decompressed using Adaptive Mixture of Independent Component Analysis (AMICA). This is the process of automatic decomposition of an adaptively learned portion of EEG data into statistically significant independent sources of brainwave activity that can be associated with identifiable dipoles [34, 61]. Adaptive Mixture of Independent Component Analysis was chosen as it uses an asymptotic Newton algorithm that has displayed more accurate ICA decomposition in static tasks and during different exercise states, including isometric muscle contractions, treadmill running, and ergometer cycling [62]. Default settings were used.

Source localisation was performed using the DIPFIT extension in EEGLAB, where dipoles were localized using the built‐in autofit function [34]. The resulting dipole location can be seen in Table 2.

Classification of independent components into what was considered significant brain components was performed using ICLabel and visual inspection. Exclusion was based on the following criteria not being met: (1) < 80% likelihood of being a brain component, (2) residual variance of < 15%, (3) dipole located in the headspace [21, 23, 63].

## Appendix B Study Limitations and Future Research Suggestions

*Study Limitations*.

Several limitations exist for the current study, most noticeably the fact that one cannot establish causality and generalizability of findings to the targeted population with one individual tested. The results gathered from this participant may have been impacted by his involvement in a treatment protocol for the persisting symptoms. Mobile EEG units provide a portable and lower‐cost solution for brainwave activity monitoring compared to stationary lab‐based EEG systems, albeit still being possibly too expensive outside of high‐performance teams and private medical care [64, 65]. Mobile EEG units also tend to have lower signal quality than clinical‐grade systems and may be more susceptible to noise, motion artifacts, and errors in electrode placement [64, 65].

The BESS test is also limited in several ways, including possible learning effects taking place between tests, the subjective scoring methods, and the test's sensitivity in detecting long‐term balance issues originating from a concussion.

Attempts to reduce the limitations of the BESS test were made via the implementation of the lumbar spine accelerometer, and for the EEG analysis, a detailed post‐processing data pipeline (Appendix A) was developed and implemented to address the potential limitations of portable EEG systems.

*Future Research*.

The need for longitudinal and comparative studies that track and compare brainwave activity in athletes who have suffered a SRC, those who are presenting with PSaC, and healthy athletes is suggested as it may allow future researchers to monitor changes in brain function over a period of time and help validate brainwave localization as a replicable biomarker in the population. In addition to this, the proposed longitudinal and comparative studies may help establish normative data for comparative analysis, and it may assist clinicians in assessing brainwave activity changes after an athlete has suffered a concussion.

It may be beneficial for future analysis to perform intra‐and inter‐group comparisons between the different stances to potentially determine the impact that stance complexity has on brainwave activity. In addition to this, it may also be of interest for future analysis to compare dipole location between the baseline tests and the balance tasks, between stances and surfaces, as well as between pathological and healthy participants.

It is further suggested that future studies set out to explore the accuracy in using mobile EEG systems combined with advanced qEEG techniques during movement‐based studies to determine regions of brain activity as well as develop normative data. This data is currently lacking.

Future research should also set out to explore in more detail the early identification and management of mental health variables in athletes, as well as the broader clinical implications and implementations for such research.
